# Short-Term Effects of Thinning on the Carbon Sink Function of Secondary Broadleaf Forest Ecosystems

**DOI:** 10.3390/plants15060868

**Published:** 2026-03-11

**Authors:** Xiaohong Wu, Xiaomei Jiang, Suyun Zheng, Weiqing Qiu, Guojun Miao, Jianjun Zhong, Lin Xu, Yongjun Shi

**Affiliations:** 1 Zhejiang Key Laboratory of Carbon Sequestration and Emission Reduction in Agriculture and Forestry, Zhejiang A&F University, Hangzhou 311300, China; wxhong@stu.zafu.edu.cn (X.W.); 15002375305@163.com (X.J.); 2 National Key Laboratory for Development and Utilization of Forest Food Resources, Zhejiang A&F University, Hangzhou 311300, China; 3 College of Environmental and Resources Science, College of Carbon Neutrality, Zhejiang A&F University, Hangzhou 311300, China; 4 Niutou Mountain Forest Farm, Suichang County, Lishui 323300, China; 18967056291@163.com (S.Z.); m13567646569@163.com (G.M.); 5 Suichang County Ecological Forestry Development Center, Lishui 323300, China; 13567646147@163.com; 6 Suichang County Hushan Forest Farm, Lishui 323300, China; 13957046353@139.com

**Keywords:** thinning intensity, forest management, greenhouse gas emissions, carbon sequestration

## Abstract

Secondary broadleaf forests constitute a vital component of China’s forest resources, characterized by diverse ecological functions, strong regeneration capacity, and widespread distribution. They possess significant potential for carbon storage, yet their carbon sink capacity is influenced by multiple factors, including successional stage, tree species composition, soil conditions, and human disturbance levels. However, the response mechanism of carbon sequestration capacity in secondary broadleaf forest ecosystems to thinning intensity remains unclear. This study aims to elucidate the effects of different thinning intensities (0% (CK), 10% (LT), 25% (MT), and 35% (HT)) on soil greenhouse gas (GHG) emissions, vegetation, and soil organic carbon sinks. Results indicate that total GHG emissions increased by 1.9%, 31.86%, and 42.18% under LT, MT, and HT, respectively. Vegetation carbon sequestration decreased by 5.26% and 16.22% under LT and MT, respectively, while increasing by 13.17% under HT. Soil organic carbon sequestration increased by 37.33% under LT, but decreased by 5.89% and 61.41% under MT and HT, respectively. In summary, compared with the control, ecosystem carbon sequestration increased by 30.66% in LT, while decreasing by 32.06% and 71.73% in MT and HT, respectively. Our study indicates that light thinning intensity can enhance the carbon sequestration potential of ecosystems and effectively mitigate climate change.

## 1. Introduction

Forests are the largest terrestrial vegetation carbon pool and carbon sink in terrestrial ecosystems [1]. As a major component of terrestrial ecosystems, they perform critically important ecological functions [2]. Carbon dioxide (CO_2_) is the most influential and longest-lasting greenhouse gas in the Earth’s climate system [3]. Due to its high emissions and significant warming effect, it plays a major role in global warming [4]. Forests possess formidable carbon sequestration capabilities, with both the vegetation carbon pool and soil carbon pool capable of storing vast amounts of carbon [5]. Forests absorb substantial CO_2_ through photosynthesis in vegetation, fixing carbon within biomass [6]. Additionally, microbial conversion and plant root systems sequester carbon in soil, where it can remain stored for decades or even centuries, playing a vital role in mitigating climate change [7,8]. Establishing sound forest management practices is crucial for alleviating climate change [9].

Common forest management techniques include reforestation, prescribed burning, and thinning [6,10]. As a prevalent forest management practice, thinning is widely applied in forest operations and management [11]. Thinning reduces competition among trees, enhances stand vitality, and thereby effectively promotes timber volume growth [12]. Appropriate thinning intensity can stimulate tree growth, improve the stand environment, and maintain ecosystem functions [13]. The benefits of thinning include the following: (1) reducing mortality rates by removing poorly growing trees, those affected by pests or diseases, or trees detrimental to overall stand growth, thereby improving forest health; (2) lowering stand density enhances stand vitality and resistance to pests and diseases; (3) increasing thinning frequency redistributes stand structure and adjusts site components, thereby improving biodiversity [12]. Following thinning, altered stand structure reduces tree density and improves understory light conditions, increasing available space for understory vegetation growth and promoting its development [14]. Furthermore, thinning alters tree biomass and respiration, leading to changes in forest ecosystem carbon dynamics [15]. However, thinning not only directly affects aboveground vegetation but also indirectly influences underground soil microbial communities and their functions through altering forest microclimates (such as light, temperature, and humidity), litter quantity and quality, root exudates, and fine root turnover [16]. Changes in these environmental factors may profoundly impact microbial community composition, metabolic activity, and the soil carbon cycling processes they mediate [17].

The carbon sequestration capacity varies among different forest types [18]. Among them, subtropical forests are particularly significant due to their high productivity, biodiversity, and carbon storage potential [19]. Subtropical evergreen broadleaf forests represent a typical vegetation type distributed across humid subtropical climatic zones [20]. Dominated by evergreen broadleaf tree species, these forests exhibit high species diversity and complex community structures, making them one of the most important ecosystems in subtropical regions [21]. Evergreen broadleaf forests represent a transitional forest type between subtropical evergreen broadleaf forests and deciduous broadleaf forests, situated within the community transition zone [22]. They exhibit rich biodiversity and high productivity, playing a primary role in ecosystem carbon storage [23,24]. Broadleaf forests are crucial for climate regulation, water conservation, biodiversity maintenance, and carbon sequestration, alongside oxygen release [25].

However, most of China’s subtropical evergreen broad-leaved forests have degraded into secondary broad-leaved forests due to long-term human disturbance or natural climate change [26]. This degradation has resulted in poor stability during succession, as well as persistent issues such as slow growth, low carbon sequestration capacity, and diminished economic value [27]. Given their extensive distribution and large area, restoring the ecological functions and economic value of secondary broad-leaved forests holds significant importance [28]. Thinning practices of varying intensities exert differing impacts on forests [29]. Implementing appropriate thinning measures to optimize stand structure can enhance the stability of secondary broadleaf forest ecosystems and boost their carbon sequestration capacity [30].

In this study, we applied different intensities of thinning measures to secondary evergreen broadleaf forests to analyze the effects of varying thinning intensities on the carbon sequestration capacity of secondary broadleaf forest ecosystems. The objectives of this study are as follows: (1) to investigate the effects of different thinning intensities on greenhouse gas fluxes and (2) to investigate the effects of different thinning intensities on the carbon sequestration capacity of secondary broadleaf forest ecosystems. We propose the following hypotheses: (1) thinning increases soil greenhouse gas emissions, with the increase being proportional to thinning intensity; (2) thinning reduces soil carbon stocks during the short recovery period by stimulating soil microbial activity; (3) heavy thinning promotes short-term vegetation carbon accumulation, but this gain may be partially offset by increased soil greenhouse gas emissions. This study comprehensively assessed the impact of thinning on ecosystem carbon sequestration functions by integrating vegetation carbon dynamics, soil carbon dynamics, soil greenhouse gas fluxes, and their driving factors. The findings provide a scientific basis for optimizing thinning techniques, enhancing carbon sequestration capacity in secondary forest management, and advancing climate change mitigation efforts.

## 2. Materials and Methods

### 2.1. Study Area

**Figure 1:**
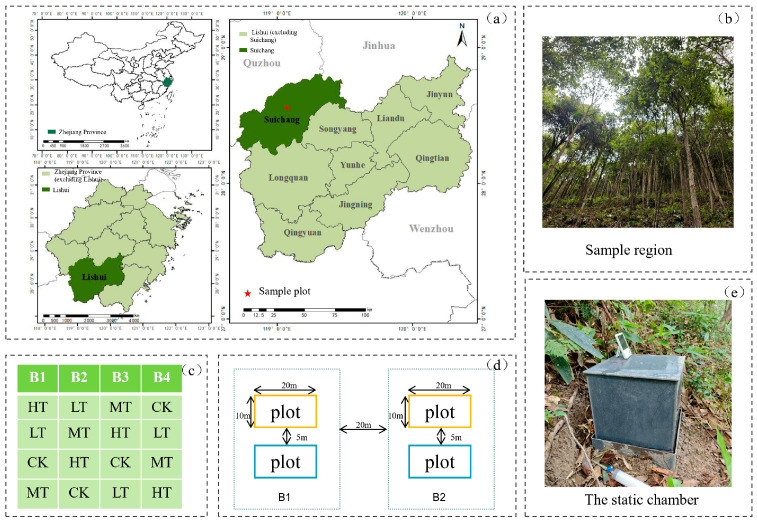
Study area location and experimental setup: (**a**) location of the study area in Suichang County, Lishui, China; (**b**) experimental area; (**c**) plot distribution map, where B denotes block (*n* = 4); (**d**) spatial relationship map of plots; (**e**) forest soil greenhouse gas monitoring collection chamber.

**Figure 2:**
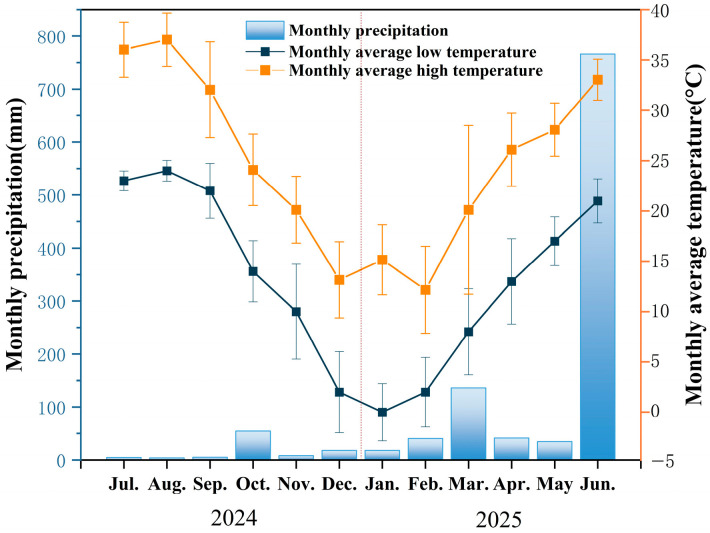
Monthly average precipitation, monthly average low temperature, and monthly average high temperature in the study area. Image data sourced from the website (https://www.tianqi24.com accessed on 30 April 2025).

The study area is located at Hushan Forest Farm in Suichang County, Lishui City, Zhejiang Province, China (119°4′ E, 28°33′ N) (Figure 1a). This region features a subtropical monsoon climate characterized by cold winters, hot summers, and distinct seasons. The area receives an average annual precipitation of 1345 mm, with an average annual temperature of 15.6 °C. The frost-free period averages 251 days annually, and the annual sunshine duration is 2146 h [31] (Figure 2). Elevations range from 400 m to 460 m. According to the 2014 FAO Soil Classification System [32], the study area’s soil is classified as Ferralsols.

### 2.2. Experimental Design

The study area is dominated by tree species such as Blue Japanese oak (*Cyclobalanopsis glauca (Thunb.) Oerst*), Chinese holly (*Ilex chinensis Sims*), and Schima (*Schima superba Gardner & Champ*). In July 2024, plots were established within secondary broadleaf forest stands sharing similar elevation, climatic conditions, and site characteristics. To reduce environmental variability and enhance precision in inter-treatment comparisons, the study area was divided into four spatially independent blocks based on similar topography, soil types, and vegetation structure, with blocks separated by at least 20 m (Figure 1c,d). Environmental conditions within each block were relatively uniform, enabling us to isolate the effects of thinning intensity from potential site variability. Within each block, four 20 m × 10 m permanent plots were randomly established and subjected to the following four thinning treatments: control (CK, 0% tree removal rate), light thinning (LT, 15%), moderate thinning (MT, 25%), and heavy thinning (HT, 35%) (Table 1). This design yielded 16 experimental plots (4 treatments × 4 blocks). To minimize interference between treatments, buffer zones at least 5 m wide were established between adjacent plots within the same block. Within each permanent plot, timber selection was based on diameter class distribution structure and tree grading. Specifically, to unlock stand growth potential and optimize intraspecific competition patterns, we identified and marked suppressed trees (primarily small-diameter classes) with poor trunk form and weakened growth vigor, as well as disruptive trees (primarily medium- and large-diameter classes) posing severe competition to surrounding target species across all diameter classes. Priority removal targeted pioneer species like sweetgum (*Liquidambar formosana Hance*), while climax species such as Blue Japanese oak (*Cyclobalanopsis glauca (Thunb.) Oerst*) and Chinese holly (*Ilex chinensis Sims*), were retained as target trees. All logging residues were cleared post-harvest to eliminate litterfall input interference on soil carbon dynamics. Following plot establishment, static chambers were deployed at the key treatment locations within each plot.

#### 2.2.1. Measurement of Soil Greenhouse Gas Emissions

From July 2024 to June 2025, greenhouse gas samples were collected monthly during sunny days in the middle of the month, between 9:00 AM and 11:00 AM. The static chamber consists of a polyvinyl chloride (PVC) base (30 cm × 30 cm × 10 cm) and a 5 mm-thick removable lid (30 cm × 30 cm × 10 cm). The upper edge of the base features a U-shaped groove (5 cm × 5 cm), while the lid has a small hole (1 cm diameter) fitted with a rubber stopper at its center. Prior to gas collection, vegetation within the base was removed using scissors. An electric fan was placed inside the base. After closing the lid, water was poured into the U-shaped groove to ensure a watertight seal. Samples are collected every 10 min (i.e., at 0, 10, 20, and 30 min) using a 100 mL syringe equipped with a three-way stopcock valve. Samples are stored in 100 mL sealed aluminum foil gas bags for laboratory analysis. Simultaneously, soil temperature at a depth of 5 cm is measured and recorded. After collecting test samples, gas samples were analyzed within 48 h using a Shimadzu gas chromatograph (GC-2014, Shimadzu Corporation, Tokyo, Japan). Sample analysis employed greenhouse gas concentration standard curves prepared under laboratory standard reference conditions (N_2_O: 5.0 × 10^−6^ mol/mol, CH_4_: 20.4 × 10^−6^ mol/mol, CO_2_: 302 × 10^−6^ mol/mol) [33].

Formula (1) calculates the fluxes of CO_2_, N_2_O, and CH_4_ [34].

(1) $$F\;=\rho \;\times \;\frac{V}{A}\;\times \;\frac{P}{P_{0}}\;\times \;\frac{dC_{t}}{d_{t}}\;\times \;\frac{T_{0}}{T}$$

where *F* represents the flux of soil CO_2_, N_2_O, and CH_4_ (CO_2_ emissions are in units of mg m^−2^h^−1^; N_2_O emissions and CH_4_ uptake are in units of μg m^−2^h^−1^); ρ denotes gas densities under standard conditions: CO_2_ = 1.98 × 10^3^ g·m^−3^, N_2_O = 1.964 × 10^3^ g·m^−3^, and CH_4_ = 7.163 × 10^2^ g·m^−3^. *A* and *V* represent the effective base area (m^2^) and volume (m^3^) of the static chamber, respectively; $\frac{P}{P_{0}}$ denotes the ratio of atmospheric pressure under standard conditions to the atmospheric pressure inside the laboratory; $\frac{dC_{t}}{d_{t}}$ indicates the linear regression slope (ppm·h^−1^) derived from the change in greenhouse gas concentrations relative to the time change inside the chamber; and $\frac{T_{0}}{T}$ represents the ratio of absolute temperature under standard conditions to the absolute temperature inside the static chamber during sampling.

Formula (2) calculates the annual greenhouse gas emissions from soil [35].

(2) $$F_{g}=\frac{\sum (F_{i+1}+F_{i})}{2}\;\times \;(t_{i+1}-t_{i})\;\times \;24\;\times {\;10}^{-5}$$

where $F_{g}$ represents the flux of soil greenhouse gases (mg·m^−2^·h^−1^), i denotes the number of samples, and t denotes the sampling time.

Formula (3) is used to calculate greenhouse gas emissions, converting the fluxes of CO_2_, N_2_O, and CH_4_ uniformly into CO_2_ equivalents [36].

(3) $${\mathrm{GWP}}_{T}=F_{{\mathrm{CO}}_{2}}+{\;298F}_{N_{2}O}\;-\;25F_{{\mathrm{CH}}_{4}}$$

where ${\mathrm{GWP}}_{T}$ denotes the total soil GWP flux (Mg CO_2_-eq ha^−1^), $F_{{\mathrm{CO}}_{2}},{\;F}_{N_{2}O}$ and $F_{{\mathrm{CH}}_{4}}$ represent the cumulative fluxes of CO_2_, N_2_O and CH_4_ respectively; 298 and 25 denote the conversion factors for N_2_O and CH_4_ fluxes to CO_2_ equivalents over a 100-year time scale [37].

#### 2.2.2. Soil Sampling and Analysis

Soil samples were collected bimonthly from July 2024 to May 2025, resulting in a total of six sampling campaigns: July 2024, September 2024, November 2024, January 2025, March 2025, and May 2025. The five-point sampling method was employed: after removing surface litter, five random points were selected within each plot to collect soil samples at depths of 0–20 cm and 20–40 cm. Soil bulk density data were simultaneously obtained using the ring-cutting method. Samples were sealed in bags and transported to the laboratory. After removing litter and residual roots from the soil, each sample is divided into two portions. One portion of fresh soil is stored at 4 °C for testing soil gravimetric water content (SWC), water-soluble organic carbon (WSOC), water-soluble organic nitrogen (WSON), microbial biomass carbon (MBC), microbial biomass nitrogen (MBN), nitrate nitrogen (NO_3_-N), and ammonium nitrogen (NH_4_-N). The other portion is air-dried for soil pH testing. To ensure consistent measurement conditions across all treatment steps and sampling time points, air-dried soil samples were used to eliminate the impact of field moisture variations on pH readings [38].

Soil gravimetric water content (SWC) data were calculated based on mass changes before and after drying samples at 105 °C [39]; soil WSOC and soil WSON data were determined using a total organic carbon analyzer (TOC-VCPH, Tokyo, Japan) according to the method of Wu et al. [40]. Soil MBC and soil MBN concentrations were determined using the chloroform fumigation method [41]. Soil nitrate nitrogen (NO_3_-N) and soil ammonium nitrogen (NH_4_-N) concentrations were determined using ultraviolet spectrophotometry and the indophenol blue colorimetric method [42]; pH values were determined using a pH meter (FE20; Mettler Toledo, Switzerland) on soil suspensions [43]; soil bulk density was measured using the ring-cutting method [44].

To determine soil organic carbon (SOC), soil samples were collected in two rounds in July 2024 and June 2025. Soil samples were randomly collected at depths of 0–20 cm and 20–40 cm within the experimental area. Soil organic carbon (SOC) was determined using the potassium dichromate external heating method, and organic carbon storage was calculated using Equation (4) [45].

(4) $$C_{\mathrm{SOC}}=\sum_{i}^{n}C_{i}\;\times \;B_{i}\;\times \;D_{i}\;\times \;(1-\omega )100^{-1}$$

where $C_{\mathrm{SOC}}$ represents the soil organic carbon stock in the 0–40 cm layer (Mg C ha^−1^), *i* denotes a specific soil layer, and $C_{i},\;B_{i}\;\mathrm{and}\;D_{i}$ represent the organic carbon content (g kg^−1^), bulk density (g m^−3^), and thickness (cm) of layer *i*, respectively, and ω depicts the mass fraction (%) of stone oak, roots, and other organisms with a diameter greater than 2 mm.

Formula (5) calculates the annual carbon sequestration rate of soil organic carbon [46].

(5) $$△\mathrm{SOC}\;=\frac{44}{12}\;\times \;(C_{\mathrm{SOC},2025}-C_{\mathrm{SOC},2024})$$

where △SOC represents the soil carbon sequestration (Mg CO_2-eq_ ha^−1^), and $C_{\mathrm{SOC},2025}$ and $C_{\mathrm{SOC},2024}$ represent the soil organic carbon stocks measured in June 2025 and July 2024, respectively (Mg C ha^−1^).

#### 2.2.3. Vegetation Carbon Stock Measurement and Calculation

In July 2024 and June 2025, individual tree measurements were conducted within 16 experimental zones, with two measurements taken during the trial period. Diameter at breast height (DBH) was measured for each tree using a caliper, and timber volume was estimated using the Zhejiang Province Timber Volume Table [47]. Forest biomass (B) was calculated using the biomass expansion factor method with Equation (6) [48] to determine the tree biomass.

(6) B =V × D × BEF × (1+R)

where B represents tree species biomass (t); *V* represents tree trunk volume (m^3^); *D* represents the basic wood density of the tree species (with bark); *BEF* denotes the biomass expansion factor for the tree species, used to convert stem volume to above-ground biomass, dimensionless; R denotes the ratio of below-ground to above-ground biomass for the tree species, dimensionless. In this study, the corresponding values are *D* = 0.598 td.m^−3^/m^3^, *BEF* = 1.674, and *R* = 0.261 [48].

Formula (7) calculates the carbon stock of forest trees.

(7) $$C_{B}=\sum B\;\times \;C_{F}\;\times \;\frac{10,000}{A_{P}}$$

where $C_{B}$ represents the carbon stock of the tree stand (Mg ha^−1^), $C_{F}$ represents the conversion coefficient between tree biomass and carbon stock ($C_{F}$ = 0.5), and $A_{P}$ represents the area of each plot (m^2^).

The annual carbon sequestration per unit area generated by trees within the plot from July 2024 to June 2025 is calculated using Formula (8).

(8) $$△C_{B}=(C_{B,2025}-C_{B,2024})\;\times \;\frac{44}{12}$$

where $△C_{B}$ represents the annual carbon sequestration per unit area of trees (Mg CO_2-eq_ ha^−1^), and $C_{B,\mathrm{year}}$ represents the carbon stock in the corresponding year (Mg ha^−1^).

#### 2.2.4. Calculation of Overall Carbon Sequestration Capacity of Vegetation and Soil Carbon Reservoirs

The overall carbon sequestration capacity is calculated using Formula (9) [46].

(9) $$C_{\mathrm{total}}=△C_{B}+{△}_{\mathrm{SOC}}-{\mathrm{GWP}}_{T}$$

where $C_{\mathrm{total}}$ represents the total carbon sink within the plot (Mg CO_2-eq_ ha^−1)^, $△C_{B}$ denotes the annual carbon sequestration by trees per unit area (Mg CO_2-eq_ ha^−1^), △SOC represents soil carbon sequestration (Mg CO_2-eq_ ha^−1^), and ${\mathrm{GWP}}_{T}$ indicates the total soil GWP flux (Mg CO_2-eq_ ha^−1^).

### 2.3. Data Analysis

All data analyzed in this study were calculated as mean values from four replicate plots per treatment (*n* = 4). Prior to analysis, the normality and homogeneity of variance for all variables were tested using SPSS 24.0 (SPSS Inc., Chicago, IL, USA). Graphical visualizations were generated using Microsoft Excel 2021, Origin 2025, and Canoco 5. To evaluate the effects of thinning intensity on soil properties (soil temperature, soil gravimetric water content, soil organic carbon, soil organic nitrogen, microbial biocarbon, microbial bio-nitrogen, nitrate nitrogen, ammonium nitrogen, and pH) and greenhouse gas fluxes (carbon dioxide, methane, nitrous oxide), single-factor analysis of variance (ANOVA) was employed followed by least significant difference (LSD) post hoc tests. To investigate the interaction between thinning intensity and month on the aforementioned variables, a two-factor ANOVA was further applied. To explore the bivariate relationships between soil properties and greenhouse gas fluxes, Pearson correlation analysis was conducted, which provided preliminary evidence for variable selection in subsequent multivariate analyses. To identify the key environmental drivers of greenhouse gas fluxes, redundancy analysis (RDA) was applied using Canoco 5. This multivariate approach allowed us to visualize and quantify the contributions of soil factors to the variation in GHG emissions across treatments and soil depths. Finally, stepwise regression analysis was performed to quantify the explanatory power of the dominant factors identified by RDA. This sequential approach—from bivariate exploration (correlation) to multivariate interpretation (RDA) to quantitative validation (regression)—provided a comprehensive framework for elucidating the mechanisms driving GHG emissions following thinning disturbance.

## 3. Results

### 3.1. Effects of Thinning on Soil Environmental Factors and Carbon and Nitrogen Pools

**Figure 3:**
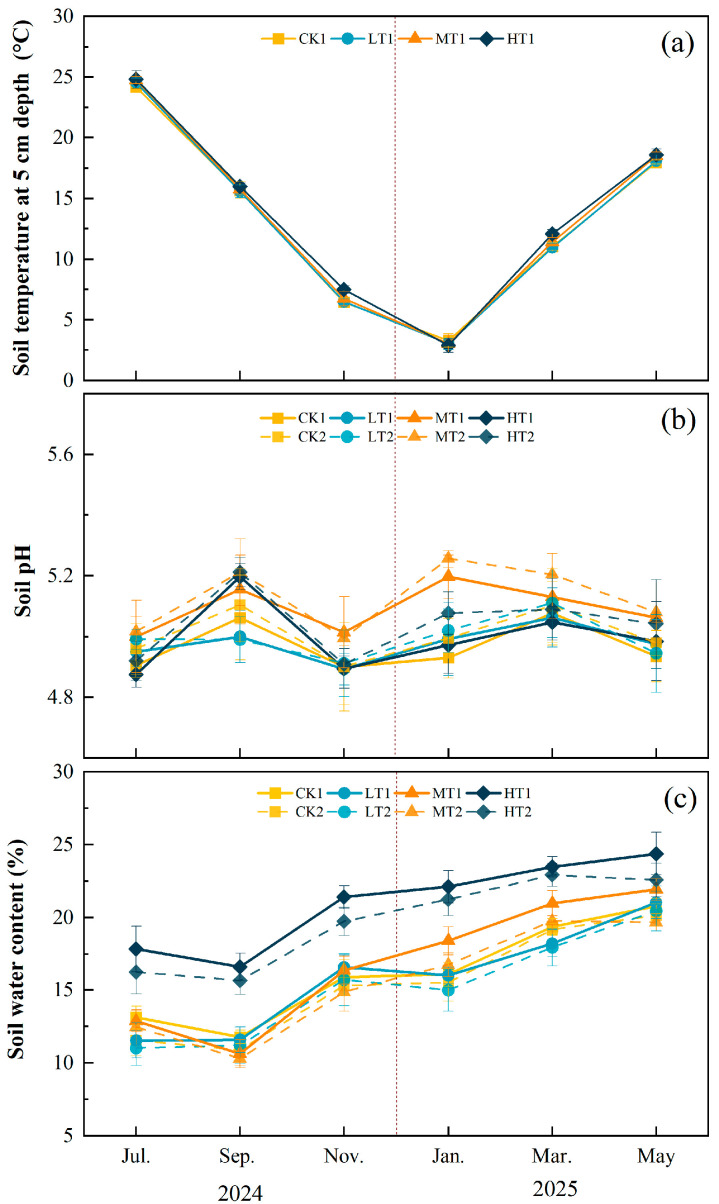
Monthly mean values (±SD, *n* = 4) of soil variables under different thinning intensities. CK, LT, MT, and HT represent control, light thinning (15%), moderate thinning (25%), and heavy thinning (35%), respectively. Suffixes 1 and 2 denote soil depths of 0–20 cm and 20–40 cm. (**a**) Soil temperature at 5 cm depth, (**b**) soil pH, (**c**) soil gravimetric water content. The vertical dashed line indicates the boundary between the 2024 (left) and 2025 (right) sampling years.

Temperature and precipitation during the experimental period (2024–2025) fell within the range of historical variability, indicating that the study was conducted under climatologically representative conditions (Tables S1 and S2). Thinning treatment had no significant effect on soil temperature at 5 cm depth (*p* > 0.05), but the month significantly influenced soil temperature (Table S3). The trends in soil temperature across treatments closely resembled the temperature trends in the study area (Figure 3a), with the lowest temperatures occurring in December–February and the highest in July–August. However, thinning significantly affected soil gravimetric water content and soil pH (*p* < 0.05). Compared to the CK treatment, LT and MT treatments did not significantly affect soil SWC, but the HT treatment significantly increased soil SWC (*p* < 0.001), with increases of 29.62% and 27.77% in the 0–20 cm and 20–40 cm soil layers, respectively (Table S3, Figure 3c). Compared with the CK treatment, soil pH significantly increased in the MT treatment (*p* < 0.01), rising by 2.41% and 2.40% in the 0–20 cm and 20–40 cm soil layers, respectively (Table S3, Figure 3b). The pH in the LT and HT treatments did not differ significantly from that in the CK treatment.

**Figure 4:**
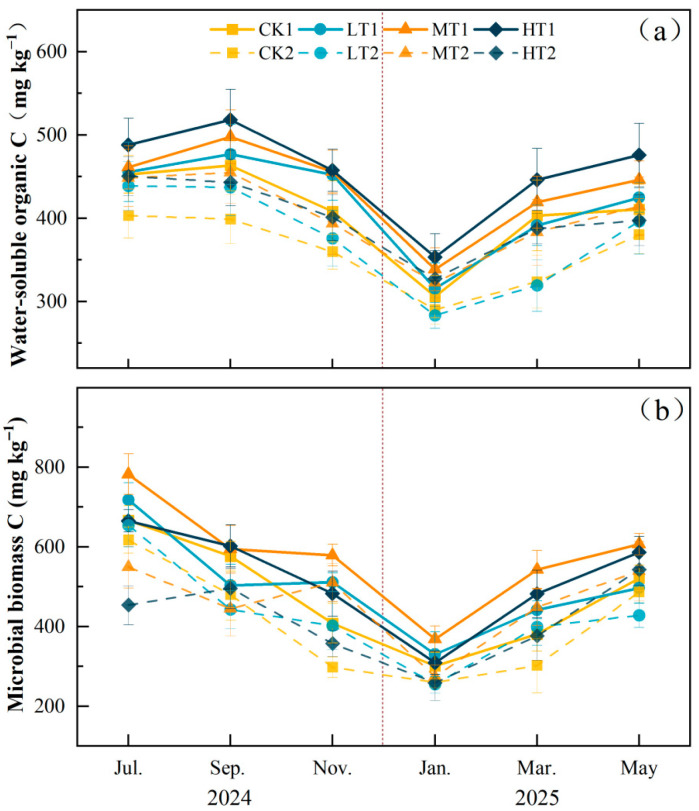
Monthly mean values (±SD, *n* = 4) of soil variables under different thinning intensities. CK, LT, MT, and HT represent control, light thinning (15%), moderate thinning (25%), and heavy thinning (35%), respectively. Suffixes 1 and 2 denote soil depths of 0–20 cm and 20–40 cm. (**a**) Soil water-soluble organic carbon, (**b**) soil microbial biomass carbon. The vertical dashed line indicates the boundary between the 2024 (left) and 2025 (right) sampling years.

In the soil carbon pool, thinning significantly affected WOSC (*p* < 0.05). Compared to CK, HT treatment exhibited significant differences in WSOC at 0–20 cm (*p* < 0.05), while the MT and HT treatments showed significant increases of 12.05% and 11.61%, respectively, in the 20–40 cm layer (*p* < 0.05). No significant differences were observed between the LT and CK treatments in either soil layer (Table S3, Figure 4a). Thinning significantly affected soil microbial biomass carbon (*p* < 0.05). In the 0–20 cm soil layer, compared with CK, LT, MT, and HT treatments increased it by 5.10%, 21.69% (*p* < 0.05), and 9.58%, respectively. In the 20–40 cm soil layer, no significant differences were observed between LT, MT, and HT treatments and CK (*p* > 0.05) (Table S3, Figure 4b).

**Figure 5:**
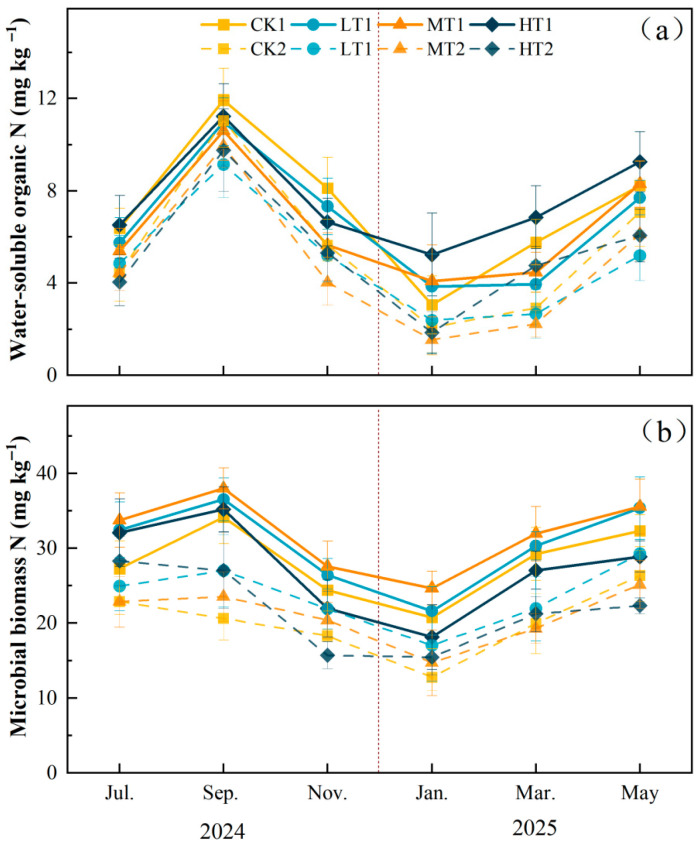
Monthly mean values (±SD, *n* = 4) of soil variables under different thinning intensities. CK, LT, MT, and HT represent control, light thinning (15%), moderate thinning (25%), and heavy thinning (35%), respectively. Suffixes 1 and 2 denote soil depths of 0–20 cm and 20–40 cm. (**a**) Soil water-soluble organic nitrogen, (**b**) soil microbial biomass nitrogen. The vertical dashed line indicates the boundary between the 2024 (left) and 2025 (right) sampling years.

**Figure 6:**
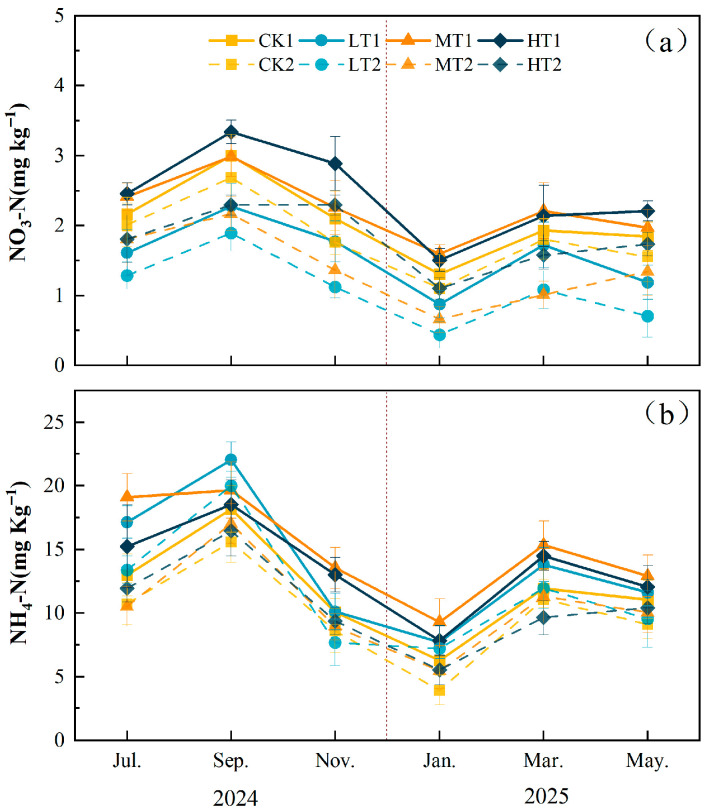
Monthly mean values (±SD, *n* = 4) of soil variables under different thinning intensities. CK, LT, MT, and HT represent control, light thinning (15%), moderate thinning (25%), and heavy thinning (35%), respectively. Suffixes 1 and 2 denote soil depths of 0–20 cm and 20–40 cm. (**a**) Soil nitrate nitrogen, (**b**) soil ammonium nitrogen.The vertical dashed line indicates the boundary between the 2024 (left) and 2025 (right) sampling years.

In the soil nitrogen pool, thinning significantly affected soil MBN in the 0–20 cm layer (*p* < 0.05). Compared with CK, MT significantly increased MBN by 13.88% (*p* < 0.05). No significant differences existed among LT, HT, and CK treatments. In the 20–40 cm layer, no significant differences in soil MBN were observed among the four treatments (Table S3, Figure 5b). Different intensities of thinning significantly affected soil nitrate nitrogen content (*p* < 0.001). Compared with CK, the monthly average NO_3_-N decreased significantly by 23.79% (*p* < 0.001). No significant differences existed among LT, MT, and HT treatments. Compared to CK, LT and MT treatments significantly reduced NO_3_-N by 40.11% and 23.63%, respectively, in the 20–40 cm soil layer (*p* < 0.001) (Table S3, Figure 6a). WSON and NH_4_-N showed no significant differences among treatments (*p* > 0.05) (Table S3, Figure 5a and Figure 6b).

### 3.2. Effects of Thinning Intensity on Soil Greenhouse Gas Emissions

**Figure 7:**
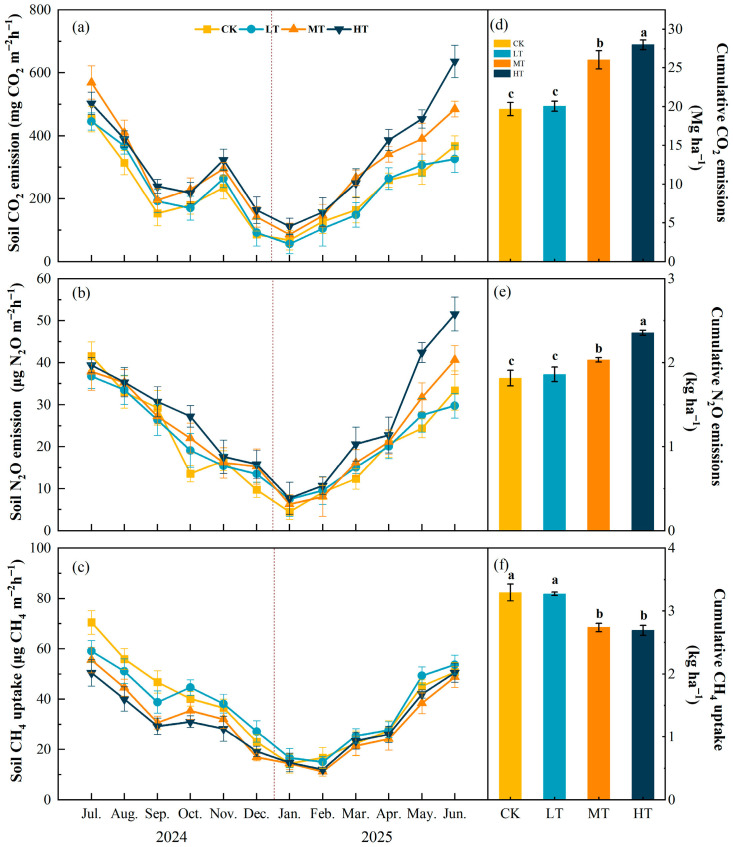
Effects of different thinning intensities on monthly greenhouse gas fluxes and annual accumulation/uptake. (**a**) Soil CO_2_ emission, (**b**) soil N_2_O emission, (**c**) soil CH_4_ uptake, (**d**) annual cumulative CO_2_ emission, (**e**) annual cumulative N_2_O emission, and (**f**) annual cumulative CH_4_ uptake. Values are presented as means ± SD (*n* = 4). Different lowercase letters above the bars indicate significant differences among treatments at *p* < 0.05.The vertical dashed line indicates the boundary between the 2024 (left) and 2025 (right) sampling years.

During the 12-month continuous monitoring experiment, thinning significantly increased CO_2_ emissions (*p* < 0.001) (Table S3, Figure 7a). Compared with CK, the monthly average soil CO_2_ emissions in MT and HT treatments showed significant increases (*p* < 0.001), rising by 33.34% and 44.63%, respectively, while no significant difference was observed between the LT treatment and CK. The annual cumulative soil CO_2_ emissions for CK, LT, MT, and HT were 19.69 ± 0.86 Mg ha^−1^, 20.07 ± 0.63 Mg ha^−1^, 26.04 ± 1.20 Mg ha^−1^, and 28.02 ± 0.64 Mg ha^−1^ (Figure 7d). LT showed no significant difference compared to CK, but MT and HT differed significantly from CK (*p* < 0.001).

Significant differences existed in N_2_O emissions among treatments. Soil N_2_O emission dynamics were generally consistent with soil CO_2_ emissions (Table S3, Figure 7b). Compared with CK, the monthly average soil N_2_O emissions increased significantly by 29.61% (*p* < 0.05), while no significant differences were observed between LT and MT treatments and CK. The annual cumulative soil N_2_O emissions for CK, LT, MT, and HT were 1.82 ± 0.09 kg ha^−1^, 1.86 ± 0.09 kg ha^−1^, 2.03 ± 0.03 kg ha^−1^, and 2.35 ± 0.03 kg ha^−1^, respectively. LT showed no significant difference compared to CK, while MT and HT differed significantly from CK (*p* < 0.001) (Figure 7e).

Under different thinning intensities, soil CH_4_ uptake exhibited consistent trends across treatments (Table S3, Figure 7c). One-way ANOVA indicated that different thinning treatments significantly affected the monthly average soil CH_4_ uptake (*p* < 0.05). However, Tukey HSD post hoc tests revealed no significant differences among treatments. The annual cumulative soil CH_4_ uptake for CK, LT, MT, and HT was 3.30 ± 0.13 kg ha^−1^, 3.28 ± 0.03 kg ha^−1^, 2.74 ± 0.07 kg ha^−1^, and 2.69 ± 0.08 kg ha^−1^, respectively. Compared with CK, LT showed no significant difference, while MT and HT showed significant differences (*p* < 0.001) (Figure 7f).

### 3.3. Effects of Soil Environmental Factors on Soil Greenhouse Gas Emissions

**Figure 8:**
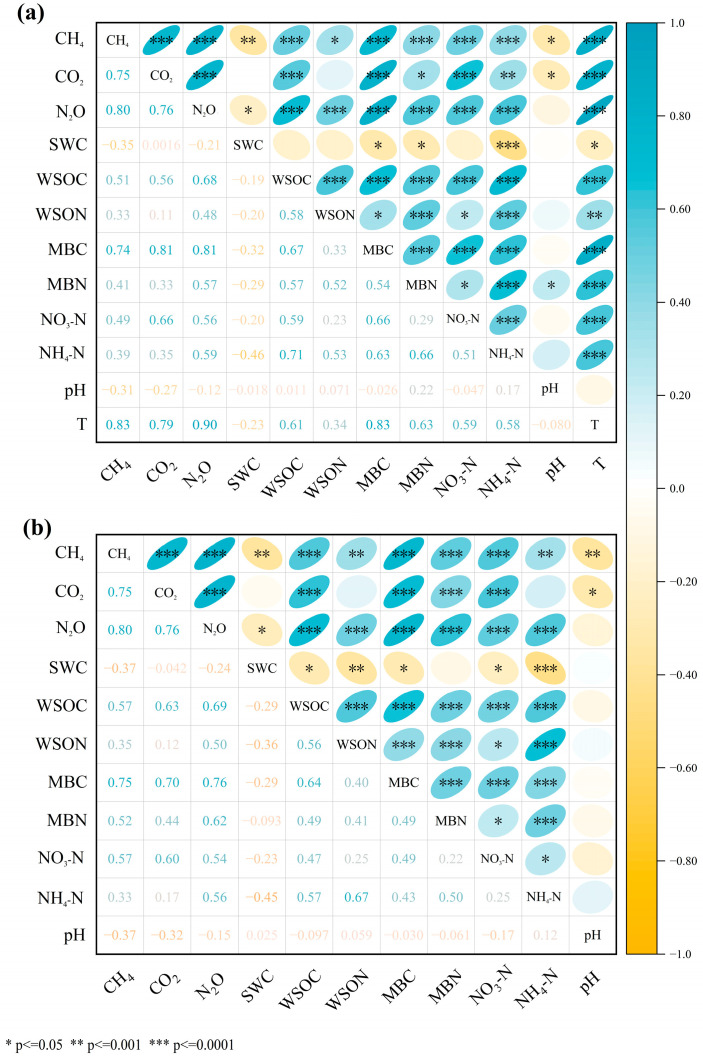
Pearson correlation analysis between soil environmental factors and greenhouse gas fluxes in secondary broad-leaved forests. Orange circles indicate positive correlations, blue circles indicate negative correlations. (**a**) 0–20 cm soil layer, (**b**) 20–40 cm soil layer.

All three greenhouse gases showed significant positive correlations with each other (r = 0.75–0.80, *p* < 0.01), indicating synchronous emission patterns (Figure 8). In the topsoil layer (0–20 cm), soil temperature (T) showed the strongest positive correlations with all greenhouse gases (r = 0.79–0.90, *p* < 0.01), followed by microbial biomass carbon (MBC) (r = 0.74–0.81, *p* < 0.01). Soil WSON showed moderate correlations (r = 0.57–0.70), particularly significant with N_2_O. Soil pH exhibited weak negative correlations (r = −0.31 to −0.12) (Figure 8a). In the deeper soil layer (20–40 cm), microbial carbon content remained the dominant factor, showing strong positive correlations (r = 0.70–0.79, *p* < 0.01), though slightly weaker than in the surface layer. pH continued to show weak negative correlations (r = −0.37 to −0.15). Notably, MBN showed moderate correlations with carbon dioxide (CO_2_) and nitrous oxide (N_2_O) (r = 0.44–0.62), with higher correlation strengths than in the topsoil. Soil moisture correlations remained weak (r = −0.37 to −0.04) (Figure 8b).

**Figure 9:**
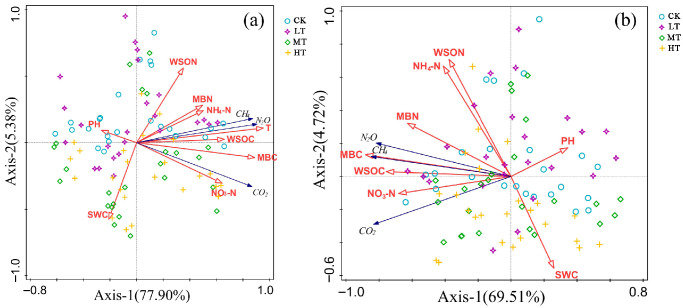
Redundancy analysis (RDA) ordination plots of soil GHG fluxes in relation to environmental factors for (**a**) 0–20 cm soil layers and (**b**) 20–40 cm soil layers.

To elucidate the driving patterns of greenhouse gas fluxes by soil physicochemical properties, redundant analysis (RDA) was employed to constrain the ordering of greenhouse gas emission data with environmental variables. Since temperature probes were deployed only at the soil surface, T was used as the representative surface soil temperature in this study. Within the 0–20 cm soil layer, RDA indicated that the variability in soil greenhouse gas fluxes was primarily driven by temperature (T) (70.7%, pseudo-F = 227, *p* < 0.01), significantly exceeding other environmental factors. pH, microbial biomass carbon (MBC), and soil gravimetric water content (SWC) acted as secondary factors, explaining 3.6%, 3.5%, and 2.9% of the variance, respectively (all *p* < 0.01), while the contributions of water-soluble organic carbon (WSOC) and microbial biomass nitrogen (MBN) were insignificant (*p* > 0.05) (Figure 9a). In the 20–40 cm soil profile, RDA revealed that microbial biomass carbon (MBC) primarily explained soil greenhouse gas flux variation (54.4%, pseudo-F = 112, *p* < 0.01), contributing 70.9% of the variance. pH (7.6%), water-soluble organic carbon (WSOC, 3.9%), and soil gravimetric water content (SWC, 1.4%) acted as secondary factors, all reaching significant levels (*p* < 0.05) (Figure 9b).

Stepwise regression analysis identified key predictors of greenhouse gas fluxes under different thinning intensities and soil depths (Table 2, Table 3 and Table 4). Results revealed distinct driving mechanisms across gases, treatments, and depths. For CH_4_, soil microbial biomass carbon (MBC) was the dominant predictor in most surface and subsurface (20–40 cm) treatments (adjusted R^2^ = 0.64–0.81). Notably, under moderate (MT) and heavy (HT) thinning in the 20–40 cm layer, NO_3_-N replaced MBC as the primary entry variable (adjusted R^2^ = 0.46–0.61), indicating that nitrogen availability becomes a key regulatory factor under high-intensity disturbance. The final models explained 71–91% of CH_4_ variance (Table 2). For CO_2_, soil temperature (T) dominated in surface control (CK1) and heavy thinning (HT1) plots (adjusted R^2^ = 0.61–0.66), while MBC dominated in surface light (LT1) and moderate (MT1) thinning plots (adjusted R^2^ = 0.74–0.78). In the subsurface layer (20–40 cm), MBC was the primary predictor in most treatments, except under MT2, where NO_3_-N again emerged as the primary predictor. Final model adjusted R^2^ values ranged from 0.72 to 0.89 (Table 3). For N_2_O, soil temperature was the core predictor across all surface treatments (0–20 cm) (adjusted R^2^ = 0.80–0.88), with final models explaining 88–93% of variance. In subsurface soils (20–40 cm), MBC and WSOC became the primary drivers, yielding final adjusted R^2^ values of 0.75–0.87 (Table 4).

### 3.4. Effects of Thinning Intensity on Stand Volume and Biomass

After 12 months of experimentation, the total tree volume accumulation within plots under CK, LT, MT, and HT treatments was 109.71 m^3^ ha^−1^, 115.07 m^3^ ha^−1^, 113.97 m^3^ ha^−1^, and 106.46 m^3^ ha^−1^, respectively (Table 5). The total volume growth rates for CK, LT, MT, and HT treatments were 9.83%, 8.80%, 7.24%, and 11.66%, respectively. Compared to CK, the HT treatment exhibited a 1.83% higher total volume growth rate, while LT and MT growth rates were both lower than CK. Total biomass and total volume growth rates were identical within plots (Table 5). The average diameter at breast height (DBH) showed no significant difference between the first and second years. However, significant differences existed in DBH growth among treatments (*p* < 0.05) (Table 5). Compared with CK, HT increased significantly by 30.61%, LT decreased by 4.82%, and MT increased by 5.13%. No significant differences were observed between LT, MT, and CK.

### 3.5. Effects of Thinning on Tree Carbon Pool and Soil Carbon Pool

The study found no significant differences in tree carbon sequestration among treatments (*p* > 0.05, Table 6). Thinning significantly affected soil carbon sequestration (Table 6). In the 0–20 cm soil layer, compared with CK, HT decreased significantly by 55.40% (*p* < 0.05), LT increased significantly by 60.31%, while MT showed no statistically significant difference from CK. In the 20–40 cm soil layer, HT significantly decreased soil carbon sequestration by 70.18% (*p* < 0.05), while LT and MT showed no statistically significant difference compared to CK. Overall, soil carbon sequestration increased significantly by 37.33% under LT and decreased significantly by 61.41% under HT (*p* < 0.05).

The GWP of GHG emissions for the CK, LT, MT, and HT treatments were 20.15 ± 0.83 Mg CO_2-eq_ ha^−1^, 20.54 ± 0.65 Mg CO_2-eq_ ha^−1^, 26.58 ± 1.90 Mg CO_2-eq_ ha^−1^, and 28.65 ± 0.63 Mg CO_2-eq_ ha^−1^, respectively. Compared to CK, the GWP of GHG emissions for LT, MT, and HT increased by 2%, 32%, and 42%, respectively. Significant differences existed among treatments (*p* < 0.05, Table 6). Among these four thinning intensities, greenhouse gas emissions showed a positive correlation with thinning intensity. In terms of total carbon sequestration, compared with CK, LT treatment increased by 30.66%, MT decreased by 32.06%, and HT significantly decreased by 71.73% (*p* < 0.05). The differences between LT and MT treatments were not significant (*p* > 0.05).

## 4. Discussion

### 4.1. Response of Soil Greenhouse Gas Emissions to Thinning Intensity

Research indicates that global warming potential (GWP) exhibits an upward trend with increasing thinning intensity, consistent with global meta-analysis findings: thinning significantly increases soil carbon dioxide and nitrous oxide emissions while reducing methane sequestration [49]. This response can be attributed to multiple interacting mechanisms, including (1) elevated soil temperatures and altered moisture conditions, stimulating microbial activity [49]; (2) altered soil priming effects mediated by shifts in microbial community structure (e.g., increased fungal-to-bacterial ratios) [50]; (3) enhanced nitrogen availability (particularly NO_3_-N), driving nitrification and denitrification processes [51]; and (4) reduced fine root biomass after tree removal, altering root-derived carbon inputs and rhizosphere processes [52].

Soil CO_2_ emissions represent the second largest terrestrial flux and constitute the primary source of greenhouse gas emissions from secondary broadleaf forest soils (Table 6), primarily originating from microbial respiration and plant root respiration [53,54]. Thinning generates substantial logging residues (e.g., branches, leaves, roots), which decompose in the plot, providing ample nutrients for soil microbial activity and thereby promoting microbial respiration [55]. As thinning intensity increases, greater soil disturbance may accelerate decomposition of existing organic matter, thereby promoting soil CO_2_ emissions [56]. Enlarged forest gaps post-thinning cause elevated soil temperatures, which accelerate microbial metabolic rates and enzyme activity. This further enhances soil organic matter decomposition, increasing soil respiration and promoting soil CO_2_ emissions [57]. Simultaneously, greater thinning intensity creates larger forest gaps, allowing more moisture to reach the ground directly and thereby increasing soil water content [58]. Higher soil water content may stimulate soil respiration, increasing CO_2_ emissions [59] (Table S3).

Soil nitrification and denitrification are primary pathways for N_2_O production, and soil N_2_O emissions are highly sensitive to changes in soil water and temperature conditions [60]. In this study, thinning treatment significantly increased soil N_2_O emission, consistent with previous research findings [61]. Pearson correlation analysis and stepwise regression analysis revealed that changes in soil active carbon, nitrogen pools, and hydrothermal conditions significantly influenced soil N_2_O emissions (Figure 8, Table 4). Post-thinning alterations in surface temperature and soil water content were observed. Previous studies indicate that increased soil moisture and temperature stimulate nitrification and denitrification rates, leading to heightened soil N_2_O emissions [62]. The observed increases in MBN and NO_3_-N concentrations following thinning may be partially attributed to the decomposition of residual roots, as root residues are known to be important sources of soil carbon and nitrogen [63]. Although direct measurements of root biomass were not performed in this study, this mechanism is consistent with previous findings in similar forest ecosystems [64]. Simultaneously, the increase in inorganic carbon pools accelerates soil nitrogen mineralization rates [65]. Second, increased soil nutrients enhance microbial activity, leading to elevated MBC concentrations and consequently promoting soil N_2_O emissions [66].

This study demonstrates that thinning treatments can significantly reduce soil CH_4_ uptake, with CH_4_ uptake negatively correlated to thinning intensity. In a thinning and replanting experiment in an eucalyptus forest, Hu et al. [61] found that during the short recovery phase following thinning and replanting, increasing thinning intensity reduced CH_4_ uptake. Forest soil CH_4_ uptake is a biological process primarily carried out by aerobic methanotrophs, which consume oxygen to oxidize CH_4_ into CO_2_ and H_2_O [67]. Thinning reduces canopy water interception, increasing surface water availability and significantly decreasing tree transpiration, leading to elevated soil water content (Table S3) and altered soil aeration [68]. Concurrently, CH_4_ uptake in secondary broadleaf forest soils decreases with increasing thinning intensity (*p* < 0.05). This may occur because, after thinning, the decomposition of dead roots remaining in the soil consumes oxygen, creating an anaerobic environment [69]. The anaerobic soil conditions inhibit the activity of aerobic methanotrophs, reducing their absorption capacity and thereby decreasing soil CH_4_ uptake [70].

### 4.2. Responses of Vegetation and Soil Carbon Sequestration to Thinning Intensity

This study found that different thinning intensities did not significantly affect carbon sequestration in the tree layer carbon pool. Carbon sequestration decreased under LT and MT treatments (Table 6), likely because thinning directly reduced biomass, leading to decreased carbon sequestration. The reduction was positively correlated with thinning intensity [71]. However, notably, tree carbon sequestration increased under the HT treatment (Table 6). This may result from altered stand structure after thinning, reducing competition among trees for light, water, and nutrients. In a thinning experiment on Fujian cypress forests, Wang et al. [72] observed that tree carbon sequestration in the canopy decreased with increasing thinning intensity in the short term after thinning. Thinning trials in secondary broadleaf forests of the Greater Khingan Range demonstrated [73] that moderate and heavy thinning increased forest carbon sequestration, while light thinning not only reduced biodiversity but also caused significant tree mortality. Therefore, thinning intensity should be tailored to specific forest stands.

Furthermore, the soil organic carbon pool constitutes a vital component of forest ecosystem carbon storage and significantly influences the carbon sequestration capacity of forest ecosystems [74]. In our study, soil SOC exhibited positive growth across all treatments; however, a nonlinear relationship was observed between thinning intensity and soil SOC increment (Table 6), consistent with findings by Gong et al. [30]. Simultaneously, with increasing soil depth, the increment of SOC in the topsoil layer (0–20 cm) exceeded that in the subsoil layer (20–40 cm) (Table 6), consistent with the findings of He et al. [75]. Moreover, in both soil layers, the increment of SOC exhibited a trend of initial increase followed by decline (LT > CK > MT > HT) (Table 6). Post-thinning, residual roots provided abundant, readily decomposable organic substrates for microorganisms. Additionally, thinning improved the microenvironment by altering soil thermal and moisture conditions, thereby promoting microbial growth and reproduction [76,77]. However, beyond a certain thinning intensity, excessive disturbance may disrupt stable microbial habitats (e.g., mycorrhizal networks). Factors such as elevated soil temperatures and increased ultraviolet radiation can reduce microbial activity [78]. Concurrently, mineralization may deplete readily decomposable carbon sources, leading to net losses in SOC [79]. Under MT and HT treatments, elevated soil MBC and greenhouse gas emissions likely stem from a positive stimulation effect: abundant fresh organic matter (e.g., root residues and litter) generated by thinning stimulates microbial communities, synergistically accelerating decomposition of pre-existing, stable SOC [80]. Concurrently, excessive thinning weakens critical underground carbon input pathways by removing large numbers of trees and causing root mortality [81]. Therefore, in MT and HT treatments, although increased understory vegetation leads to higher carbon inputs [82], these inputs may be quantitatively insufficient to compensate for carbon losses due to enhanced microbial activity, or qualitatively consist of easily decomposable carbon that struggles to form stable SOC [83]. This short-term imbalance between carbon inputs and outputs ultimately manifests as a significant reduction in net SOC accumulation, thereby weakening the soil carbon sink function under HT treatments [81,84]. Although this study did not directly measure microbial community structure, extensive research indicates that forest thinning significantly alters microbial communities [85].

### 4.3. Limitations and Future Research Directions

Although MBC has been identified as a primary driver of greenhouse gas emissions, microbial community composition has not been directly measured. Future studies combining high-throughput sequencing or phospholipid fatty acid analysis will help elucidate the mechanistic link between microbial community changes and greenhouse gas emissions. Furthermore, this study examined only the short-term effects of thinning. Long-term monitoring is needed to validate the temporal consistency of observed patterns. Future studies should also incorporate measurements of soil enzyme activity (e.g., urease, cellulase) and soil carbon components (e.g., particulate organic carbon, dissolved organic carbon) to deeply analyze the mechanisms underlying thinning-induced changes in soil carbon cycling. Filling these knowledge gaps will advance our understanding of how thinning influences forest carbon sink function.

## 5. Conclusions

Consistent with H1, soil GHG emissions increased with thinning intensity, with CO_2_ and N_2_O showing the strongest responses. Supporting H2, thinning reduced soil carbon stocks during the short-term recovery period, with enhanced microbial activity identified as the key mechanism. In line with H3, heavy thinning promoted short-term vegetation carbon accumulation, but this gain was partially offset by increased soil GHG emissions.

Therefore, regarding the impact of forest management practices on forest carbon sequestration, the LT treatment proved most effective for short-term stand carbon sequestration. Although the HT treatment achieved the highest vegetation carbon sequestration, it resulted in a significant reduction in soil carbon sequestration. From the perspective of timber management and utilization, the HT treatment most effectively promoted timber accumulation in the short term. However, this study only conducted one year of monitoring and recording, so the results can only reflect short-term impacts. Additionally, this study did not include data on the carbon pool of understory vegetation and litter, so the results may contain some errors. Therefore, to better understand the impact of thinning treatments on the carbon sink function of secondary broad-leaved forests, it is necessary to conduct long-term monitoring and more comprehensive carbon pool indicator measurements.

## Figures and Tables

**Table 1:** Stand characteristics and thinning intensity parameters across four treatments. CK: control group, LT: light thinning group, MT: moderate thinning group, HT: heavy thinning group, DBH: diameter at breast height. Values are presented as means ± standard deviation (SD) (*n* = 4).

Treatment	Altitude (m)	Aspect	Slope (°)	Pre-Harvest (Trees·ha^−1^)	Post-Harvest (Trees·ha^−1^)	Basal Area Removal (%)	Initial DBH (cm)	After DBH (cm)
CK	448 ± 13	Southeast	44 ± 2	2550 ± 612	2550 ± 612	0	10.45 ± 1.68	10.45 ± 1.68
LT	445 ± 11	Southeast	41 ± 1	2712 ± 425	2287 ± 342	31.79 ± 3.47	11.06 ± 0.78	11.57 ± 0.40
MT	437 ± 10	South	38 ± 3	2500 ± 663	1875 ± 513	23.36 ± 1.83	11.65 ± 0.78	12.78 ± 0.80
HT	430 ± 7	Southwest	30 ± 3	2150 ± 346	1387 ± 201	14.18 ± 3.84	11.88 ± 2.45	13.51 ± 2.51

**Table 2:** Stepwise regression model of soil CH_4_ uptake. CK, LT, MT, and HT represent thinning intensities of 0%, 10%, 25%, and 35%, respectively. The numbers 1 and 2 following the treatment codes denote the soil layers: 1 (0–20 cm) and 2 (20–40 cm). This study measured soil CH_4_ uptake, soil microbial biomass carbon (MBC), soil pH, soil ammonium nitrogen (NH_4_-N), soil temperature (T), soil water-soluble organic carbon (WSOC), soil nitrate nitrogen (NO_3_-N), soil microbial biomass nitrogen (MBN), and soil water-soluble organic nitrogen (WSON). Standardized coefficients are used as the model coefficients. Degrees of freedom: 24; *** indicate significance at *p* < 0.001.

GHG	Treatment	Model	df	R^2^	*p*
CH_4_	CK1	Y = 0.904MBC	24	0.81	***
		Y = 0.911MBC − 0.185pH	24	0.838	***
	LT1	Y = 0.864T	24	0.736	***
		Y = 0.678T + 0.326WSOC	24	0.801	***
	MT1	Y = 0.877MBC	24	0.759	***
		Y = 0.537MBC + 0.401T	24	0.796	***
	HT1	Y = 0.870T	24	0.745	***
		Y = 0.861T − 0.285pH	24	0.822	***
	CK2	Y = 0.899MBC	24	0.8	***
		Y = 0.911MBC + 0.232NH_4_-N	24	0.849	***
	LT2	Y = 0.807MBC	24	0.635	***
		Y = 0.503MBC + 0.415WSOC	24	0.705	***
	MT2	Y = 0.792NO_3_-N	24	0.61	***
		Y = 0.515NO_3_-N + 0.436MBC	24	0.716	***
		Y = 0.578NO_3_-N + 0.590MBC − 0.398NH_4_-N	24	0.838	***
		Y = 0.480NO_3_-N + 0.526MBC − 0.471NH_4_-N − 0.285pH	24	0.882	***
		Y = 0.449NO_3_-N + 0.418MBC − 0.465NH_4_-N − 0.298pH + 0.203MBN	24	0.91	***
	HT2	Y = 0.695NO_3_-N	24	0.459	***
		Y = 0.475NO_3_-N + 0.419MBC	24	0.573	***
		Y = 0.399NO_3_-N + 0.521MBC − 0.434pH	24	0.759	***
		Y = 0.440NO_3_-N + 0.753MBC − 0.325pH − 0.383WSON	24	0.823	***

**Table 3:** Stepwise regression model of soil CO_2_ emission. CK, LT, MT, and HT represent thinning intensities of 0%, 10%, 25%, and 35%, respectively. The numbers 1 and 2 following the treatment letters denote the 0–20 cm and 20–40 cm soil layers, respectively. Regression models for soil CO_2_ emissions include soil temperature (T), soil microbial biomass nitrogen (MBN), soil ammonium nitrogen (NH_4_-N), soil pH, soil water-soluble nitrogen (WSON), soil microbial biomass carbon (MBC), soil nitrate nitrogen (NO_3_-N), and soil gravimetric water content (SWC). Standardized coefficients are used as the model coefficients. Degrees of freedom: 24; *** indicate significance at *p* < 0.001.

GHG	Treatment	Model	df	R^2^	*p*
CO_2_	CK1	Y = 0.701T	24	0.611	***
		Y = 0.998T − 0.400MBN	24	0.721	***
		Y = 1.077T − 0.517MBN + 0.337NH_4_-N	24	0.826	***
		Y = 1.057T − 0.475MBN + 0.324NH_4_-N − 0.194pH	24	0.861	***
		Y = 1.095T − 0.367MBN + 0.422NH_4_-N − 0.199pH − 0.242WSON	24	0.888	***
	LT1	Y = 0.886MBC	24	0.775	***
		Y = 1.002MBC + 0.259SWC	24	0.822	***
	MT1	Y = 0.869MBC	24	0.743	***
		Y = 0.948MBC − 0.293WSON	24	0.819	***
		Y = 0.612MBC − 0.337WSON + 0.410T	24	0.862	***
	HT1	Y = 0.823T	24	0.662	***
		Y = 0.812T − 0.381pH	24	0.805	***
	CK2	Y = 0.729MBC	24	0.511	***
		Y = 0.735MBC − 0.365pH	24	0.634	***
		Y = 0.453MBC − 0.380pH + 0.454NO_3_-N	24	0.761	***
	LT2	Y = 0.827MBC	24	0.669	***
		Y = 0.820MBC − 0.250pH	24	0.722	***
	MT2	Y = 0.734NO_3_-N	24	0.518	***
		Y = 0.492NO_3_-N − 0.408pH	24	0.613	***
		Y = 0.546NO_3_-N − 0.543pH − 0.330NH_4_-N	24	0.685	***
		Y = 0.370NO_3_-N − 0.455pH − 0.416NH_4_-N + 0.413MBC	24	0.773	***
	HT2	Y = 0.658MBC	24	0.407	***
		Y = 0.734MBC − 0.528pH	24	0.679	***
		Y = 0.980MBC − 0.428pH − 0.372WSON	24	0.732	***
		Y = 0.868MBC − 0.382pH − 0.417WSON + 0.261NO_3_-N	24	0.775	***

**Table 4:** Stepwise regression model of soil N_2_O emission. CK, LT, MT, and HT represent thinning intensities of 0%, 10%, 25%, and 35%, respectively. The numbers 1 and 2 following the treatment letters denote the 0–20 cm and 20–40 cm soil layers, respectively, for establishing soil N_2_O emissions with soil temperature (T), soil gravimetric water content (SWC), soil ammonium nitrogen (NH_4_-N), soil microbial biomass carbon (MBC), soil nitrate nitrogen (NO_3_-N), soil water-soluble organic nitrogen (WSON), soil water-soluble organic carbon (WSOC), soil microbial biomass nitrogen (MBN), and soil pH. Standardized coefficients are used as the model coefficients. Degrees of freedom: 24; *** indicate significance at *p* < 0.001.

GHG	Treatment	Model	df	R^2^	*p*
N_2_O	CK1	Y = 0.901T	24	0.804	***
		Y = 0.860T − 0.213SWC	24	0.842	***
		Y = 0.874T − 0.195SWC + 0.182NH_4_-N	24	0.872	***
	LT1	Y = 0.895T	24	0.792	***
		Y = 0.807T + 0.280WSON	24	0.86	***
		Y = 0.760T + 0.237WSON − 0.201SWC	24	0.893	***
	MT1	Y = 0.940T	24	0.878	***
		Y = 0.884T + 0.170WSON	24	0.9	***
		Y = 0.638T + 0.172WSON + 0.289MBC	24	0.922	***
	HT1	Y = 0.916T	24	0.832	***
		Y = 0.841T + 0.205WSON	24	0.864	***
		Y = 0.909T + 0.236WSON + 0.222SWC	24	0.906	***
	CK2	Y = 0.847MBC	24	0.704	***
		Y = 0.665MBC + 0.291NO_3_-N	24	0.747	***
	LT2	Y = 0.816WSOC	24	0.651	***
		Y = 0.502WSOC + 0.429MBC	24	0.728	***
		Y = 0.374WSOC + 0.417MBC + 0.303MBN	24	0.798	***
		Y = 0.298WSOC + 0.341MBC + 0.384MBN − 0.240SWC	24	0.837	***
		Y = 0.380WSOC + 0.573MBC + 0.289MBN − 0.279SWC − 0.353NO_3_-N	24	0.875	***
	MT2	Y = 0.697MBC	24	0.463	***
		Y = 0.473MBC + 0.465WSOC	24	0.619	***
		Y = 0.680MBC + 0.449WSOC − 0.410NH_4_-N	24	0.747	***
	HT2	Y = 0.809MBC	24	0.638	***
		Y = 0.603MBC + 0.367MBN	24	0.722	***
		Y = 0.630MBC + 0.384MBN − 0.256pH	24	0.782	***

**Table 5:** Effects of different thinning intensities on tree growth.Different lowercase letters in the same row indicate significant differences among treatments at the 0.05 level (LSD test, *n* = 4).

Treatment	Time	DBH (cm)	Diameter at DBH (cm)	Total Growing Stock (m^3^ ha^−1^)	Total Biomass (t ha^−1^)	Biomass Growth Rate (%)
CK	2025.06	10.45 ± 1.68	0.42 ± 0.06 b	109.71	138.49	9.83%
	2024.07	10.87 ± 1.72		99.89	126.10	
LT	2025.06	11.57 ± 0.40	0.40 ± 0.05 ab	115.07	145.25	8.80%
	2024.07	11.97 ± 0.41		105.76	133.51	
MT	2025.06	12.78 ± 0.80	0.44 ± 0.05 ab	113.97	143.86	7.24%
	2024.07	13.23 ± 0.77		106.27	134.15	
HT	2025.06	13.51 ± 2.51	0.55 ± 0.11 a	106.46	134.38	11.66%
	2024.07	14.06 ± 2.61		95.34	120.35	

**Table 6:** Changes in carbon sequestration among different carbon pools under various treatments. ΔSOC1: carbon sequestration in the 0–20 cm soil layer; ΔSOC2: carbon sequestration in the 20–40 cm soil layer. Different lowercase letters within the same row indicate significant differences at the 0.05 level (LSD test, *n* = 4).

	CK	LT	MT	HT
Tree carbon sequestration	22.73 ± 6.35 a	21.53 ± 4.10 a	19.04 ± 10.70 a	25.72 ± 10.71 a
ΔSOC1	21.04 ± 6.33 b	33.73 ± 4.86 a	20.91 ± 3.11 b	9.38 ± 2.88 c
ΔSOC2	14.43 ± 5.19 a	14.98 ± 5.60 a	12.47 ± 2.93 ab	4.30 ± 2.50 b
Soil carbon sequestration	35.47 ± 7.87 b	48.71 ± 4.56 a	33.38 ± 3.60 b	13.69 ± 5.15 c
Cumulative soil CO_2_ emission	19.69 ± 0.86 c	20.07 ± 0.63 c	26.04 ± 1.20 b	28.02 ± 0.64 a
Cumulative soil N_2_O emission	0.54 ± 0.03 c	0.55 ± 0.03 c	0.61 ± 0.01 b	0.70 ± 0.01 a
Cumulative soil CH_4_ uptake	0.08 ± 0.01 a	0.08 ± 0.01 a	0.07 ± 0.01 b	0.07 ± 0.01 b
GWP of GHG emissions	20.15 ± 0.83 c	20.54 ± 0.65 c	26.58 ± 1.90 b	28.65 ± 0.63 a
Total carbon sequestration	38.04 ± 2.91 ab	49.71 ± 8.60 a	25.85 ± 9.45 b	10.75 ± 5.69 c

## Data Availability

The original contributions presented in this study are included in the article/Supplementary Material. Further inquiries can be directed to the corresponding authors.

## Supplementary Materials

The following supporting information can be downloaded at: https://www.mdpi.com/article/10.3390/plants15060868/s1, Table S1. Monthly precipitation and monthly average high and low temperatures in the experimental area from 2020 to 2023. Table S2. Annual precipitation, annual average high temperature, annual average low temperature, and extreme values for the experimental area from 2020 to 2023. Table S3. Results of one-way ANOVA for soil GHG fluxes and physicochemical properties under different thinning intensities in a secondary broadleaf forest results of the two-way (thinning intensity, soil layer, and their interaction).
