# Supplementary material for: Short-Term Effects of Thinning on the Carbon Sink Function of Secondary Broadleaf Forest Ecosystems
Source: Plants (Basel). 2026 Mar 11;15(6):868. doi: 10.3390/plants15060868 (PMC13029957; doi:10.3390/plants15060868)
Supplement: Supplementary file 1 [file plants-15-00868-s001.zip › plants-4139781-supplementary.pdf]

Supplementary Materials

Table S1. Monthly precipitation and monthly average high and low temperatures in the experimental area from 2020 to 2023.

	T_Avg_High(°C)				T_Avg_Low(°C)				Prep_Total(mm)			
	2020	2021	2022	2023	2020	2021	2022	2023	2020	2021	2022	2023
Jan.	-	14	11	13	-	1	4	2	-	12	42	37.9
Feb.	20	18	11	14	10	7	2	4	0	70.3	98.1	150
Mar.	19	19	22	19	10	10	10	8	0	122.4	214.3	54.6
Apr.	23	23	24	24	10	13	12	13	63.4	83.7	231.6	328.3
May	30	28	25	28	19	18	15	16	274	469.2	146.1	258.8
Jun.	31	31	31	31	23	21	21	21	272.7	274.1	700.2	509.2
Jul.	33	34	37	34	25	24	24	23	185.9	94.3	129.4	244
Aug.	35	33	37	33	25	23	24	22	9.7	222.4	79.1	226.5
Sep.	28	33	32	32	18	22	19	21	153.8	150.4	64.3	385.4
Oct.	24	25	25	25	13	16	13	15	14	118.4	3	55.4
Nov.	21	18	22	20	10	8	12	8	9.9	77.2	16.6	182.9
Dec.	12	15	11	15	3	3	2	3	21.6	11.4	54	15.3

Note: T_Avg_High denotes average high temperature, T_Avg_Low denotes average low temperature, Prep_Total denotes total precipitation.

Table S2. Annual precipitation, annual average high temperature, annual average low temperature, and extreme values for the experimental area from 2020 to 2023.

	T_Avg_High (°C)	T_Avg_Low (°C)	T_Ext_High (°C)	T_Ext_Low (°C)	Prep_Total(mm)

2020	25	15	38	-6	1005.1
Ext_Date			(07/19)	(12/30)	
2021	24	13	37	-6	1705.9
Ext_Date			(07/14)	（01/01)	
2022	24	13	41	-4	1778.6
Ext_Date			(08/23)	(12/19)	
2023	24	13	39	-7	2448.3
Ext_Date			(07/11)	(12/23)	

Note: T_Avg_High denotes average high temperature, T_Ext_High denotes extreme maximum temperature, T_Ext_Low denotes extreme minimum temperature, Ext_Date denotes date of extreme value, Prep_Total denotes total precipitation.

Table S3. Results of one-way ANOVA for soil GHG fluxes and physicochemical properties under different thinning intensities in a secondary broadleaf forest results of the two-way (thinning intensity,soil layer,and their interaction) .

	Soil depth （cm）	CK	LT	MT	HT	One-Way ANOVA (thinning intensity)	Two-way ANOVA		
							Thinning intensity	month	T×M
**CO_2 emission ($mg\ m^{-2}\ h^{-1}$)**	0	222.27±13.55c	227.79±7.60bc	296.37±13.69ab	321.46±10.21a	***	***	***	***
**N_2O emission ($\mu g\ m^{-2}\ h^{-1}$)**	0	20.70±1.31b	21.20±0.96ab	23.16±0.31ab	26.83±0.32a	*	**	***	***
**CH_4 Uptake ($\mu g\ m^{-2}\ h^{-1}$)**	0	37.46±1.56a	37.23±0.30a	31.04±0.77a	30.79±0.96a	*	***	***	***
**soil temperature (°C)**	5	13.11±0.24a	13.12±0.18a	13.38±0.24a	13.64±0.24a	ns	***	***	ns
**SWC(%)**	0-20	16.17±0.51b	15.83±0.22b	16.87±0.55b	20.96±0.46a	***	***	***	**
	20-40	15.45±0.33b	15.23±0.49b	15.65±0.14b	19.74±0.11a	***			

**pH**	0-20	4.97±0.06b	4.98±0.03b	5.09±0.05a	5.00±0.05b	**	***	***	ns
	20-40	5.01±0.06b	5.00±0.03b	5.13±0.04a	5.04±0.05ab	**			
**WSOC(mg kg^{-1})**	0-20	407.34±7.38b	419.40±15.09ab	441.15±11.46b	452.40±16.00a	*	***	***	ns
	20-40	359.54±5.29b	375.19±12.87ab	402.88±20.02a	401.27±11.84a	*			
**WSON(mg kg^{-1})**	0-20	7.25±0.46a	6.60±0.63a	6.54±0.26a	7.63±0.10a	ns	***	***	ns
	20-40	5.54±0.22a	4.91±0.51a	4.72±0.36a	5.30±0.55a	ns			
**MBC(mg kg^{-1})**	0-20	475.90±24.37b	500.19±21.94ab	579.12±5.16a	521.48±8.29ab	*	***	***	***
	20-40	406.99±13.58a	430.32±5.88a	461.18±17.27a	413.96±14.94a	ns			
**MBN(mg kg^{-1})**	0-20	28.02±0.55ab	30.44±2.43ab	31.91±1.37a	27.22±1.26b	*	***	***	ns
	20-40	20.15±0.57b	23.69±0.53a	21.00±1.78ab	21.76±0.98ab	ns			
**NO_3-N(mg kg^{-1})**	0-20	2.06±0.19a	1.57±0.16b	2.24±0.03a	2.42±0.07a	***	***	***	*
	20-40	1.82±0.15a	1.09±0.09b	1.39±0.07b	1.84±0.11a	***			
**NH_4-N(mg kg^{-1})**	0-20	11.75±0.67b	13.74±0.68ab	14.98±0.98a	13.53±0.47ab	ns	***	***	**
	20-40	9.84±0.69a	11.63±1.12a	10.54±1.22a	10.55±0.73a	ns			

Note: CK, LT, MT, and HT represent thinning intensities of 0%, 10%, 25%, and 35%, respectively.Soil temperature was measured in situ at 5 cm depth . Soil pH and other chemical properties were determined based on samples collected at 0 – 20 cm and 20 – 40 cm depths. SWC, soil gravimetric water content; WSOC, water-soluble organic carbon; WSON, water-soluble organic nitrogen; MBC, microbial biomass carbon; MBN, microbial biomass nitrogen; NO_3-N, nitrate nitrogen; NH_4-N, ammonium nitrogen,T×M, thinning intensity/month interaction. Values are presented as means ± standard deviation (SD)(n=4). Different lowercase letters within the same row indicate significant differences among thinning intensities based on the least significant difference (LSD) test at P<0.05, Ns,*,**,*** indicate no significant difference, P<0.05, P<0.01, P<0.001, respectively.
